# Effects of Monolaurin on Oral Microbe–Host Transcriptome and Metabolome

**DOI:** 10.3389/fmicb.2018.02638

**Published:** 2018-11-06

**Authors:** Viviam de Oliveira Silva, Luciano José Pereira, Silvana Pasetto, Maike Paulino da Silva, Jered Cope Meyers, Ramiro Mendonça Murata

**Affiliations:** ^1^Department of Veterinary Medicine, Federal University of Lavras, Lavras, Brazil; ^2^Division of Periodontology, Diagnostic Sciences, Dental Hygiene and Biomedical Science, Herman Ostrow School of Dentistry, University of Southern California, Los Angeles, CA, United States; ^3^Department of Health Sciences, Federal University of Lavras, Lavras, Brazil; ^4^Department Foundational Sciences, School of Dental Medicine, East Carolina University, Greenville, NC, United States; ^5^Department of Microbiology and Immunology, Brody School of Medicine, East Carolina University, Greenville, NC, United States

**Keywords:** *Aggregatibacter actinomycetemcomitans*, periodontal disease, immune system, fibroblast, keratinocyte, monolaurin

## Abstract

The aim of this *in vitro* study was to evaluate the effects of monolaurin against *Aggregatibacter actinomycetemcomitans* (Aa) and determine their effects on the host transcriptome and metabolome, using an oral cell/bacteria co-culture dual-chamber model to mimic the human periodontium. For this, the Aa, was applied to cross the monolayer of epithelial keratinocytes (OBA-9) to reach the fibroblasts layer (HGF-1) in the basal chamber. The Monolaurin treatments (25 or 50 μM) were added immediately after the inoculation of the dual-chamber with Aa. After 24 h, the transcriptional factors and metabolites produced were quantified in the remaining cell layers (insert and basal chamber) and in supernatant released from the cells. The genes IL-1α, IL-6, IL-18, and TNF analyzed in HGF-1 concentrations showed a decreased expression when treated with both concentration of Monolaurin. In keratinocytes, the genes IL-6, IL-18, and TNF presented a higher expression and the expression of IL-1α decreased when treated with the two cited concentrations. The production of glycerol and pyruvic acid increased, and the 2-deoxytetronic acid NIST, 4-aminobutyric acid, pinitol and glyceric acid, presented lower concentrations because of the treatment with 25 and/or 50 μM of Monolaurin. Use of monolaurin modulated the immune response and metabolite production when administered for 24 h in a dual-chamber model inoculated with *A. actinomycetemcomitans*. In summary, this study indicates that monolaurin had antimicrobial activity and modulated the host immune response and metabolite production when administered for 24 h in a dual-chamber model inoculated with *A. actinomycetemcomitans*.

## Introduction

Periodontal disease is characterized by bacterial infection associated with the presence of biofilm, resulting in chronic inflammation of the tooth supporting tissues leading to a progressive destruction of periodontal tissue and alveolar bone. Signs and symptoms of periodontitis can include gingival swelling, bleeding during brushing, periodontal pockets, increased spacing between the teeth and tooth loss (Irfan et al., 2001; Michaud et al., 2007; Savage et al., 2009; Gurav, 2014).

The most common form of periodontal disease, denominated periodontitis, affects approximately 50% of the adult population with its severe forms affecting 10–15% of these individuals (Chapple and Genco, 2013).

The oral biofilm is composed of at least 800 different species of bacteria (Paster et al., 2006; Filoche et al., 2010). Numerous periodontopathogenic bacteria (*Porphyromonas gingivalis, Fusobacterium nucleatum, Streptococcus intermedius*, and others) are responsible for the initiation and maintenance of periodontal inflammation (Coussens and Werb, 2002; Oringer, 2002; Loos, 2005; Moutsopoulos and Madianos, 2006; de Almeida et al., 2007). The *Aggregatibacter actinomycetemcomitans* is a fermentative, gram-negative and capnophilic coccobacillus. It is not only considered the most important agent of localized aggressive periodontitis lesions, but is associated with chronic periodontitis as well (Slots et al., 1980; Zambon et al., 1983; Haraszthy et al., 2000; Gafan et al., 2004; Yang et al., 2004; Cortelli et al., 2009; Rakić et al., 2010; da Silva-Boghossian et al., 2011). The progression of periodontal disease is associated with the virulence factors of the microorganism, together with the vulnerability of the host. Aa produces leukotoxin, adhesins, bacteriocins, and lipopolysaccharide, which are responsible for interacting with the host cells triggering an inflammatory response in the periodontium (Cortelli et al., 2009). In addition, another critical factor for soft tissue inflammation and bone resorption is the immune-inflammatory host response to the bacterial biofilm (Salvi and Lang, 2005).

According to Aimetti (2014), non-surgical periodontal treatments possess limitations, such as the long-term maintainability of deep periodontal pockets, risk of disease recurrence, and skill of the operator. The development of new therapeutic agents that have the ability to inhibit the biofilm formation and modulate the inflammatory response can have a major impact in the prevention and treatment of periodontal disease. Monolaurin, also known as glycerol monolaurate or lauroyl, is a natural surfactant compound commonly used in cosmetics and the food industry. It is recognized by the Food and Drug Administration (FDA) as a GRAS (Generally Recognized as Safe) food additive since 1977. This natural compound is found in coconut oil and human breast milk (Fu et al., 2006; Peterson and Schlievert, 2006).

Monolaurin presents antibacterial and antiviral activity *in vitro* (Projan et al., 1994; Isaacs, 2001; Preuss et al., 2005; Peterson and Schlievert, 2006; Carpo et al., 2007), which may be of great interest in the treatment and/or prevention of various infections. However, the effect of monolaurin on periodontopathogenic bacteria has yet to be determined.

Therefore, the aims of this *in vitro* study were to evaluate the cytotoxicity, the antimicrobial effect of monolaurin against *A. actinomycetemcomitans* and to determine its effect on the host transcriptome and metabolome using a gum cell – bacteria co-culture model.

## Materials and Methods

### Monolaurin

For carrying out the experiments, the monolaurin utilized was 1-lauroyl-rac-glycerol (Sigma-Aldrich, St. Louis, MO, United States). For dilution, sterilized deionized water was used as the vehicle.

### Cells and Bacterial Strain

Human gingival fibroblast – HGF-1 (ATCC^®^ CRL-2014) were cultured in Dulbecco’s Modified Eagle’s Medium (DMEM) (Lonza, Walkersville, MD, United States) with 10% Fetal Bovine Serum (FBS) (Lonza, Walkersville, MD, United States). Human gingival epithelial cells (keratinocyte OBA-9) were cultured in specific medium for keratinocytes, Defined Keratinocyte-SFM (Life Technologies, Carlsbad, CA, United States) (Oda and Watson, 1990; Kusumoto et al., 2004).

*Aggregatibacter actinomycetemcomitans* (strain D7S-1) was cultivated from a subgingival plaque from an African-American female patient diagnosed with generalized aggressive periodontitis (Chen et al., 2010). The bacteria were grown in Trypticase Soy Broth (TSB) (Becton Dickinson, Franklin Lakes, NJ, United States).

### Antimicrobial Activity

Monolaurin antimicrobial activity was evaluated in *A. actinomycetemcomitans* after 24 h of treatment. Microorganisms were inoculated [1 × 10^6^ colony-forming unit per milliliter (CFU/mL) in a 96-well microtiter plate with TSB (Becton Dickinson, Franklin Lakes, NJ, United States)] and monolaurin was immediately added in various concentrations (0.5–1,000 μM) to determine the minimum inhibitory concentration (MIC) (Branco-de-Almeida et al., 2011). Microplates were maintained in a humidified incubator at 37°C and 5% CO_2_. After incubation, bacterial growth was assayed by measurement of absorbance at 660 nm. MIC was defined as the lowest concentration of monolaurin that had restricted growth to a level, 0.05 at 660 nm (no visible growth).

### Cytotoxicity Assay

*In vitro* cytotoxicity effect was measured by the fluorometric resazurin method (O’Brien et al., 2000). OBA-9 or HGF-1 cells were seeded (1 × 10^5^ cells/mL) in 96-well microtiter plates and maintained in a humidified incubator at 37°C and 5% CO_2_. After 24 h, cell morphology was observed under an inverted microscope (EVOS FL Life Technologies, Carlsbad, CA, United States) to confirm that they had adhered to the bottom of each well and were presenting proper morphology. The monolaurin (0.5–1,000 μM) was added to the cell cultures and incubated at 37°C and 5% CO_2_. After 24 h, the medium was discarded, cells were washed with room temperature phosphate buffered saline (PBS) (Lonza, Walkersville, MD, United States), and fresh, room temperature medium was added with resazurin (Cell Titer Blue Viability Assay – Promega Corp., Madison, WI, United States). Subsequently, the plate was incubated at 37°C and 5% CO_2_. After 4 h, the contents of the wells were transferred to a new microplate and the fluorescence was read in a microplate reader (SpectraMax M5, Molecular Devices, Sunnyvale, CA, United States) with excitation 550 nm, emission 585 nm, and cut off 570 nm (Pasetto et al., 2014). To calculate the results and obtain the LD_50_ (Lethal Dose), we performed a non-linear regression the program MasterPlex ReaderFit (Hitachi Solutions America).

### Dual-Chamber Model

A dual-chamber, oral cell/bacteria co-culture was used to investigate the immunological effects of monolaurin (Silva et al., 2017). HGF-1 cells (1 × 10^5^) were seed in the basal chamber of a 24-well plate. Transwell inserts (8 μm pore × 0.3 cm^2^ of culture surface – Greiner Bio-One, Monroe, NC, United States) were positioned in each well and OBA-9 cells (1 × 10^5^) were seeded into the inserts. DMEM (Lonza, Walkersville, MD, United States) with 10% FBS (Lonza, Walkersville, MD, United States) was used for the medium. The plates were incubated at 37°C in humid air containing 5% CO_2_ for 24 h (Silva et al., 2017). Trans Epithelial Electric Resistance (TEER) of each cell layer was measured with a Millicell-ERS Volt-Ohm Meter (Millipore, Bedford, MA, United States). Cell layer confluence in the Transwell insert was measured daily until reaching optimal TEER (>150 Ohm/cm^2^) after 48 h. Once reaching optimal TEER, the media of the basal chamber and the insert were replaced with new media containing *A. actinomycetemcomitans* (1 × 10^6^ CFU/mL). Medium containing the microorganism was added to the insert, passing through the upper layer of cells (OBA-9) and reaching the bottom cell layer (HGF-1). Immediately after the inoculation of oral cell/bacteria co-culture with *A. actinomycetemcomitans* the monolaurin treatments (25 and 50 μM) were added and the plate was incubated at 37°C in humid air containing 5% CO_2_. The time of exposure of the microorganism to monolaurin was 24 h. Each experiment was repeated three times with two replicates per group (*n* = 6). The experiment was divided in three groups: (1) Control – oral cell/bacteria co-culture with Aa inoculated and no treatment; (2) Mono 25 – oral cell/bacteria co-culture with Aa inoculated and treated with 25 μM of monolaurin; (3) Mono 50 – oral cell/bacteria co-culture with Aa inoculated and treated with 50 μM of monolaurin.

### Collecting Samples for Analysis

After the treatment period, liquid contents from each well was collected and centrifuged at 1,200 rpm for 10 min. Following centrifugation, supernatant was divided into two aliquots for enzyme-linked immunosorbent assay (ELISA) and metabolome analysis. RNA was isolated from the remaining cell layers of HGF-1 and OBA-9 (surface of the wells and inserts) for gene analysis in quantitative real-time PCR.

### ELISA Assay

Inflammatory cytokines were determined using a commercial ELISA (Multi-Analyte ELISArray – Qiagen, Valencia, CA, United States). The cytokines were measured by standard ELISA protocol using a panel of 12 inflammatory cytokines: interleukin-1 alpha (IL-1α), interleukin-1 beta (IL-1β), interleukin-2 (IL-2), interleukin-4 (IL-4), interleukin-6 (IL-6), interleukin-8 (IL-8), interleukin-10 (IL-10), interleukin-12 (IL-12), interleukin-17 alpha (IL-17α), interferon gamma (IFNγ), tumor necrosis factor alpha (TNFα), Granulocyte-Macrophage Colony Stimulating Factor (GM-CSF). Cytokines that presented the greatest change in concentration were selected for detailed analysis. These cytokines were analyzed using a standard ELISA protocol (Single Analyte ELISA Kit – Qiagen, Valencia, CA, United States).

### Gene Expression – Quantitative Real-Time PCR

Total RNA was extracted through a commercial RNA purification kit (Mini Kit Qiagen RNeasy Protocol – Qiagen, Valencia, CA, United States) and the purity and quantity were measured in a NanoPhotometer P360 (Implen, Westlake Village, CA, United States). Total RNA was converted into single-stranded cDNA using a high-capacity reverse transcription kit (QuantiTec Reverse Transcription Kit – Qiagen, Valencia, CA, United States). An array for evaluation of gene expression of the inflammatory response was completed by quantitative real-time PCR (Prime PCR Pathway Plate/Acute Inflammation Response – Bio-Rad, Hercules, CA, United States) from the cDNA obtained. Based on these results, six genes/primers were selected for detailed study: interleukin 1 alpha (IL-1α), interleukin 6 (IL-6), interleukin 18 (IL-18), caspase 3 (CASP3), matrix metallopeptidase 1 (MMP-1), and tumor necrosis factor (TNF) (QuantiTect Primer Assay – Qiagen, Valencia, CA, United States). QuantiTect SYBR Green PCR Kits (Qiagen, Valencia, CA, United States) were used to determine the gene expression of the selected primers. The reaction product was quantified by relative quantification using Glyceraldehyde 3-phosphate dehydrogenase (GAPDH) as the reference gene. Data were analyzed as fold-change compared to control. The cycle threshold was calculated and interpreted using CFX Connect (Bio-Rad, Hercules, CA, United States) (Pfaffl, 2001).

### Metabolome Analysis

Samples for metabolome analysis were treated as described by Silva et al. (2017) and analyzed at the West Coast Metabolomics Center (UC Davis Genome Center, Davis, CA, United States) by means of gas chromatography/mass spectrometry (Agilent 6890, Santa Clara, CA/Leco Pegasus IV, St. Joseph, MI, United States). Then, the metabolites were compared with the standard library for proper identification (Fiehn and Kind, 2007).

### Statistical Analysis

Data were expressed as mean ± standard deviation. The comparison between the groups was made through analysis of variance (ANOVA) and when a difference was determined, the Bonferroni test was applied. Data were analyzed using GraphPad Prism (version 5.01, GraphPad Software, San Diego, United States). The level of significance was set at *p* < 0.05.

## Results

### Antimicrobial Activity and Cytotoxicity Assay

**FIGURE 1:**
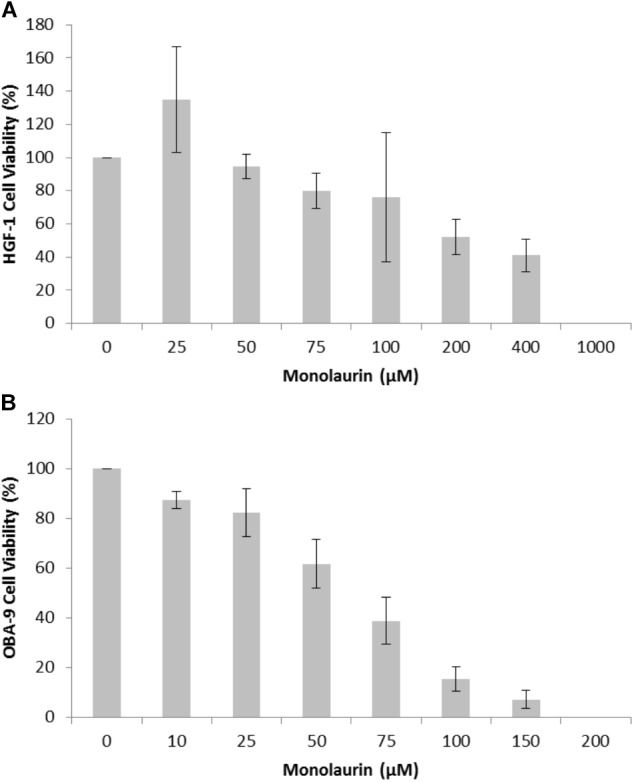
Cytotoxicity assay of cells treated with different doses of monolaurin in an oral cell/bacteria co-culture inoculated with *A. actinomycetemcomitans*: **(A)** HGF-1 cell (fibroblasts); **(B)** OBA-9 cell (keratinocytes). Cell viability was presented in percentage (%) ± standard deviation. Control group = 100% viability, *n* = 6. Non-treated cells were considered as 100% viability; DMSO was used as positive control to demonstrate an appropriate system-cell response (100% cytotoxicity – data not shown).

In order to determine antimicrobial activity of monolaurin, a range of 0.5–1,000 μM was tested. Antibacterial activity was detected at 25–50 μM. The minimum inhibitory concentration (MIC) was established between 25 and 50 μM. The cytotoxicity assays were conducted in HGF-1 and OBA-9 cells and the results are shown in Figure 1. For HGF-1 the LD_50_ was 146 μM of monolaurin and for OBA-9 the LD_50_ was 69 μM of monolaurin.

### ELISA

**FIGURE 2:**
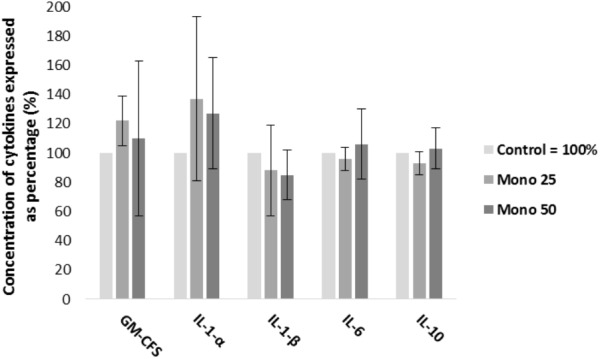
Percent concentrations of GM-CSF, IL-1α, IL-1-β, IL-6, and IL-10 by ELISA, obtained from cell culture supernatant. Oral cell co-culture inoculated with *A. actinomycetemcomitans* and treated with 25 μM of monolaurin (Mono 25) or 50 μM of monolaurin (Mono 50) compared with the control group. Control group is oral cell co-culture inoculated with *A. actinomycetemcomitans* and no treatment. The control group mean is expressed as 100% and treated groups have their mean relative to the control group. Mean ± standard deviation; *n* = 4.

Enzyme-linked immunosorbent assay (ELISA) results are presented in Figure 2. Both treatments (25 and 50 μM) of monolaurin increased the concentrations of GM-CSF and IL-1α. The concentrations of IL-6 and IL-10 increased only with the 50 μM treatment. On the other hand, IL-1β decreased as a function of monolaurin.

### Gene Expression – Quantitative Real-Time PCR

**FIGURE 3:**
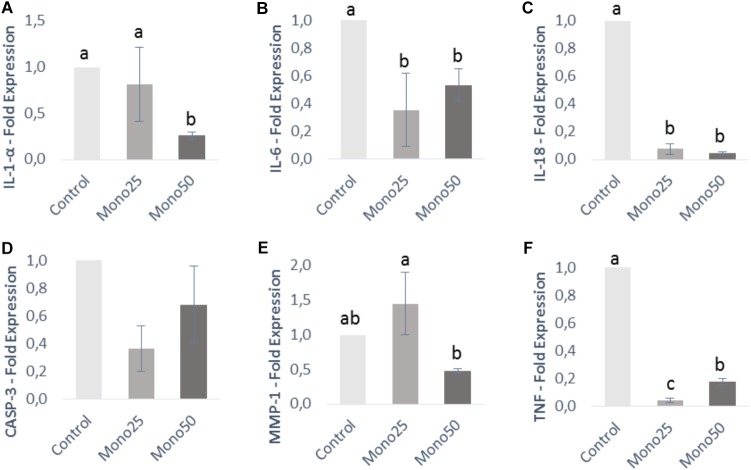
Relative expression of genes from HGF-1 cells (fibroblasts) by quantitative PCR: **(A)** IL-1α; **(B)** IL-6; **(C)** IL-18; **(D)** CASP3; **(E)** MMP-1; **(F)** TNF. Oral cell/bacteria co-culture inoculated with *A. actinomycetemcomitans* and treated with different doses of monolaurin (25 μM – Mono 25 or 50 μM – Mono 50). Control group is oral cell co-culture inoculated with *A. actinomycetemcomitans* and no treatment. The control group has their mean expressed equal to 1 and treated groups have their mean relative to the control group. Different letters (a, b, and c) indicate statistical difference between groups. The results were expressed by mean ± standard deviation; *n* = 6 and *p* < 0.05.

**FIGURE 4:**
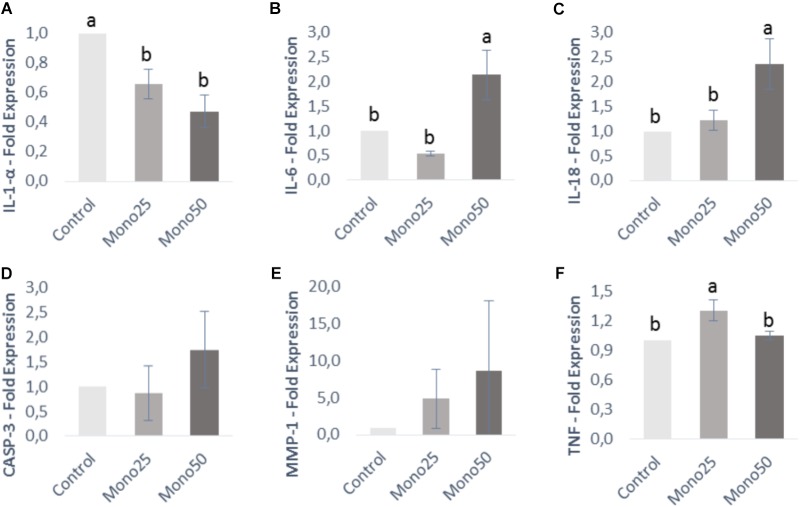
Relative expression of genes from OBA-9 cells (keratinocytes) by quantitative PCR: **(A)** IL-1α; **(B)** IL-6; **(C)** IL-18; **(D)** CASP3; **(E)** MMP-1; **(F)** TNF. Oral cell/bacteria co-culture inoculated with *A. actinomycetemcomitans* and treated with different doses of monolaurin (25 μM – Mono 25 or 50 μM – Mono 50). Control group is oral cell co-culture inoculated with *A. actinomycetemcomitans* and no treatment. The control group has their mean expressed equal to 1 and treated groups have their mean relative to the control group. Different letters (a, b, and c) indicate statistical difference between groups. The results were expressed by mean ± standard deviation; *n* = 6 and *p* < 0.05.

Quantitative real-time PCR results are presented in Figures 3, 4. The genes IL-1α, IL-6, IL-18, and TNF analyzed in HGF-1 showed a decreased expression (*p* < 0.05) when treated with 25 or 50 μM of monolaurin compared with group Control (Figures 3A–C,F). CASP3 and MMP-1 genes showed no difference (Figures 3D,E).

Keratinocytes presented a higher IL-6, IL-18, and TNF gene expression (*p* < 0.05) when treated with 25 or 50 μM of monolaurin compared with the control group (Figures 4B,C,F). IL-1α gene decreased expression (*p* < 0.05) in the group treated with both concentrations (Figure 4A). CASP-3 and MMP-1 genes showed no difference (Figures 4D,E).

### Metabolome

**FIGURE 5:**
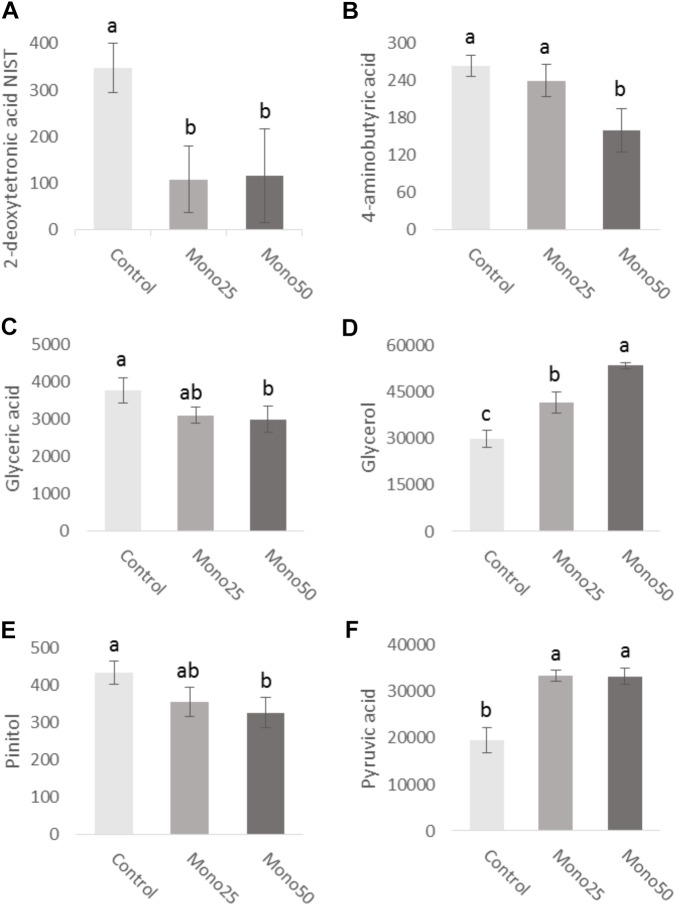
Metabolites obtained from cell culture supernatant (HGF-1 and OBA-9 co-culture cells): **(A)** 2-deoxytetronic acid NIST; **(B)** 4-aminobutyric acid; **(C)** glyceric acid; **(D)** glycerol; **(E)** pinitol; **(F)** pyruvic acid. Oral cell/bacteria co-culture model inoculated with *A. actinomycetemcomitans* and treated with different doses of monolaurin (25 μM – Mono 25 or 50 μM – Mono 50). Control group is oral cell co-culture inoculated with *A. actinomycetemcomitans* and no treatment. Different letters (a, b, and c) indicate statistical difference between groups. The data is expressed in relative peak heights (mAU) from HPLCMS analysis, which are unitless (mean ± standard deviation); *n* = 4 and *p* < 0.05.

The metabolome study returned a total of 283 metabolites. Of these, 120 were identified and only 6 presented a statistical difference as a function of monolaurin treatment. Some metabolites presented significantly altered concentrations (Figure 5). It was observed that both treatments of monolaurin increased the production of glycerol (Figure 5D) and pyruvic acid (Figure 5F) when compared to control. On the other hand, the 2-deoxytetronic acid NIST (Figure 5A) showed the opposite result, lower concentrations of this metabolite, in function of the treatment with 25 and 50 μM of monolaurin. The 50 μM monolaurin treatment group presented lower concentrations of 4-aminobutyric acid (Figure 5B), glyceric acid (Figure 5C), and pinitol (Figure 5E), against the control group.

## Discussion

Periodontal disease is known to be a widespread oral health problem across the world. Several methods for prevention and treatment of oral diseases have been used over time and new technologies are being developed. However, little attention has been given to the understanding of how periodontal structures are affected and how these structures react to different types of treatments (Balloni et al., 2016). Currently, some mouthwashes and toothpastes have been used as topical antimicrobial agents (Barnett, 2003). However, monolaurin is not listed as an active ingredient in commercially available mouthwashes. Monolaurin has been shown to possess diverse biological activities, such as antibacterial and antiviral properties (Preuss et al., 2005; Li et al., 2009; Tangwatcharin and Khopaibool, 2012; Seleem et al., 2016). In our *in vitro* study, susceptibility assays of monolaurin showed inhibition of microorganism grow at 25–50 μM with minimal cytotoxicity. The putative pathways by which monolaurin affects Aa may involve: (i) increased membrane permeability and cell lysis, (ii) disruption of electron transport chain and uncoupling oxidative phosphorylation, and (iii) inhibition of membrane enzymatic activities and nutrient uptake (Yoon et al., 2018). In addition, co-culture supernatant were assessed for expression of host pro-inflammatory cytokines, gene expression of inflammatory cytokines and metabolomic profile in human cells infected with *A. actinomycetemcomitans*.

The gingival tissue comprises the oral epithelium and superficial underlying connective tissue. These tissues are sites of initiation for inflammatory processes and the first to be affected by biofilm (Balloni et al., 2016). Gingival epithelial cells represent a physical barrier against bacteria and are involved in processes of innate immunity (Jang et al., 2015). Keratinocytes and fibroblasts represent the major cell types present in gingival epithelial tissues. They interact directly with pathogenic microorganisms and are able to express a variety of cytokines and chemokines such as IL-1α, IL-6, IL-8, TNF, among others (Bodet et al., 2007; Groeger and Meyle, 2015). In the present study, there was a decrease in the gene expression of IL-1α, IL-6, and IL-18 and TNF in HGF-1 and an increase in the expression of IL-6, IL-18, and TNF in OBA-9 cells treated with monolaurin indicating a positive modulation in the inflammatory response of these cells.

Interleukin-1 alpha is a polypeptide that plays several roles in tissue homeostasis, immunity and has a proinflammatory nature. It is produced mainly by macrophages and monocytes, but also epithelial cells and fibroblasts (Champagne et al., 2003; Balloni et al., 2016). IL-1α plays a critical role in protecting the body against invaders, such as bacteria and viruses, and is involved in extracellular matrix and bone metabolism (bone resorption, disintegration, and removal of bone tissue that is no longer needed). Therefore, IL-1α is considered a marker of periodontal disease (Govindarajan et al., 2015; Groeger and Meyle, 2015).

Here, we observed a decrease in the expression of IL-1α by HGF-1 and OBA-9 cells in the monolaurin-treated groups which may be related to a beneficial inflammatory response since, elevated levels of IL-1α and IL-1β in the gingival crevicular fluid are commonly found in patients with periodontal disease. This increased production of IL-1 is associated with a higher inflammatory response to the bacterial stimulus, resulting in more severe disease as well as an unfavorable response to treatments (Kornman et al., 1997; Engebretson et al., 1999; McGuire and Nunn, 1999; Shirodaria et al., 2000).

Interleukin-6 is associated with acute phase reactions and is usually secreted concomitantly with other proinflammatory cytokines. Some authors relate this cytokine to the destruction of periodontal tissue by favoring the accumulation of inflammatory cells and by activating and releasing inflammatory mediators that in turn accentuate the response to periodontal disease (de Lima Oliveira et al., 2012; Zhang et al., 2016).

The kinetics of production of IL-18 against the action of bacteria is rapid, according to its proinflammatory nature. It plays a central role in systemic and local inflammation (Biet et al., 2002). One study evaluated IL-18 levels in crevicular gingival fluid in four groups of patients: healthy periodontium, gingivitis, chronic periodontitis, and aggressive periodontitis. The results demonstrated that IL-18 concentration in the crevicular gingival fluid was low in the group with healthy periodontium and progressively increased in groups from gingivitis, to aggressive periodontitis, and finally chronic periodontitis. As the inflammation increased, there was a concomitant increase in IL-18 level (Nair et al., 2016). In the current study, treatment with monolaurin decreased the IL-18 gene expression in fibroblasts, however, an opposite result was observed in keratinocytes, where IL-18 expression was increased in the group treated with 50 μM monolaurin.

Tumor necrosis factor plays a central role in periodontitis, being associated with the disease because of its ability to induce destruction of connective tissue and its effects on bone resorption through the activation of osteoclasts. Thus, TNF is one of the first proinflammatory cytokines produced in response to infection by pathogenic bacteria (Stashenko et al., 1987; de Lima Oliveira et al., 2012; Zhang et al., 2016). Here, the authors observed a reduction in the gene expression of TNF in monolaurin treated fibroblasts. On the other hand, in keratinocytes, TNF expression was higher in cells treated with 25 μM of this compound. Animal models challenged with *P. gingivalis* were treated with antimicrobial drugs (chlorhexidine, minocycline, and doxycycline) in order to evaluate its effect on the inflammatory response. Minocycline induced higher levels of TNF while chlorhexidine reduced TNF levels, this result led the authors to believe that these agents modified the inflammatory response to *P. gingivalis* regardless of its antimicrobial effect, each in its own way (Houri-Haddad et al., 2008). Overall, monolaurin modulate the expression of inflammatory cytokines, suggesting it may have a modulatory role on the host pro-inflammatory response to help eradicate the bacterial infection. Similarly, previous studies suggested that monolaurin play a significant role on T cell functions and signaling by altering TCR-induced LAT, PLC-γ, and AKT cluster formation PI3K-AKT signaling axis, and calcium influx which ultimately decrease the cytokine production (Zhang et al., 2016).

The knowledge of the metabolic profile can contribute to understanding of the periodontal disease course as well as identify possible metabolites as biomarkers. This study evaluated the effects caused by monolaurin on the cellular metabolome and found significant variations. Relative quantification revealed lower levels of 2-deoxytetronic acid NIST, 4-aminobutyric acid, glyceric acid and pinitol, and higher levels of glycerol and pyruvic acid in cells treated with 25 or 50 μM monolaurin. Analysis of fibroblast metabolome (HGF) treated with IL-1β in combination with titanium dioxide nanoparticles (TiO_2_ NPs), demonstrated that IL-1β induction reduced concentrations of primary metabolites, especially those of urea cycle, polyamine, S-adenosylmethionine and glutathione synthetic pathways. The addition of TiO_2_ NPs further increased these metabolic changes induced by IL-1β. These findings may be useful for the future establishment of new metabolic markers and therapeutic strategy for gingival inflammations (Garcia-Contreras et al., 2015). One study compared the salivary metabolic profile of healthy patients and patients with periodontal disease. Changes in several classes of metabolites have been observed in individuals with periodontal disease. According to the authors, such changes reflected an increase in host–bacterial interactions in the diseased state (Barnes et al., 2011).

## Conclusion

In summary, this study indicates that monolaurin possesses antimicrobial activity and modulates the host immune response and modulates production when administered for 24 h in a dual-chamber model inoculated with *A. actinomycetemcomitans*. These findings demonstrate that monolaurin could be considered a potential candidate for *in vivo* studies, which may translate into its potential clinical use to treat pathogenic microbe–host interactions.

## Author Contributions

VS conceived and designed the experiments, performed the experiments, analyzed the data, performed the statistical analysis, and wrote the paper. LP conceived and designed the experiments, contributed to reagents, materials, and analysis tools, analyzed and critically reviewed the data. MS and SP performed the experiments and analyzed the data. JM analyzed the data, and critically reviewed and interpreted the data. RM conceived and designed the experiments, contributed to reagents, materials, and analysis tools, performed the experiments, analyzed and critically reviewed the data. All authors contributed to manuscript revision, read, and approved the submitted version.

## Conflict of Interest Statement

The authors declare that the research was conducted in the absence of any commercial or financial relationships that could be construed as a potential conflict of interest.
