# Supplementary material for: Effects of Monolaurin on Oral Microbe–Host Transcriptome and Metabolome
Source: Front Microbiol. 2018 Nov 6;9:2638. doi: 10.3389/fmicb.2018.02638 (PMC6237204; doi:10.3389/fmicb.2018.02638)
Supplement: Supplementary file 1 [file Table_1.DOCX]

Supplementary Table 1. Metabolites obtained from cell culture supernatant (HGF-1 and OBA-9 co-culture cells) Oral cell/bacteria co-culture model inoculated with *A. actinomycetemcomitans* and treated with different doses of monolaurin (25 µM - Mono 25 or 50 µM - Mono 50). The data is expressed in relative peak heights (mAU) from HPLCMS analysis, which are unitless.

| BinBase Name | Ret.index | Quant mz | BB id | KEGG | PubChem | Control | | Monolaurin 50 µM | | Monolaurin 25 µM | |
| --- | --- | --- | --- | --- | --- | --- | --- | --- | --- | --- | --- |
|  |  |  |  |  |  | Average | St Dev | Average | St Dev | Average | St Dev |
| xylulose NIST | 553450 | 173 | 31632 | C00312 | 439205 | 371 | 253 | 601 | 417 | 608 | 136 |
| xylose | 546699 | 307 | 231 | C00181 | 135191 | 1789 | 676 | 2195 | 1483 | 1703 | 813 |
| xylitol | 567437 | 217 | 5857 | C00379 | 6912 | 137 | 19 | 198 | 22 | 154 | 47 |
| valine | 313502 | 144 | 3 | C00183 | 6287 | 862386 | 72044 | 834947 | 63422 | 857133 | 43383 |
| urea | 332913 | 189 | 22329 | C00086 | 1176 | 10151 | 1348 | 9721 | 2587 | 9872 | 3349 |
| uracil | 385735 | 241 | 1664 | C00106 | 1174 | 834 | 832 | 1308 | 1061 | 1368 | 1418 |
| tyrosol | 510842 | 179 | 100888 | C06044 | 10393 | 48 | 19 | 39 | 14 | 42 | 9 |
| tyrosine | 671252 | 218 | 16 | C00082 | 6057 | 79390 | 2784 | 78595 | 7124 | 75301 | 7872 |
| tryptophan | 780482 | 202 | 14 | C00078 | 6305 | 41641 | 896 | 48957 | 3277 | 44612 | 5786 |
| trans-4-hydroxyproline | 484934 | 140 | 97 | C01157 | 5810 | 112173 | 3542 | 117801 | 6332 | 118449 | 9375 |
| thymine | 420133 | 255 | 1692 | C00178 | 1135 | 1588 | 53 | 1767 | 51 | 1592 | 115 |
| threonine | 409568 | 218 | 26 | C00188 | 6288 | 99129 | 2830 | 99365 | 6727 | 96583 | 4742 |
| threonic acid | 497572 | 292 | 172 | C01620 | 5460407 | 7372 | 1401 | 7449 | 1799 | 7072 | 1856 |
| tagatose | 627414 | 307 | 16813 | C00795 | 439312 | 169 | 68 | 130 | 63 | 122 | 40 |
| sucrose | 915139 | 271 | 173 | C00089 | 5988 | 1589 | 322 | 1663 | 702 | 1671 | 313 |
| succinic acid | 370608 | 247 | 161 | C00042 | 1110 | 39131 | 7666 | 49982 | 7444 | 44789 | 11937 |
| stearic acid | 787622 | 117 | 13 | C01530 | 5281 | 57398 | 22008 | 63306 | 11222 | 71081 | 27815 |
| shikimic acid | 611100 | 204 | 286 | C00493 | 8742 | 260 | 61 | 502 | 101 | 494 | 212 |
| serine | 395020 | 218 | 25 | C00065 | 5951 | 176254 | 164656 | 20186 | 19713 | 68353 | 76435 |
| saccharic acid | 699211 | 333 | 11214 | C00818 | 33037 | 205 | 18 | 241 | 25 | 215 | 30 |
| ribose | 553135 | 217 | 1662 | C00121 | 5779 | 380 | 116 | 423 | 119 | 364 | 153 |
| ribonic acid | 599680 | 292 | 1683 | C01685 | 5460677 | 348 | 77 | 192 | 45 | 206 | 91 |
| ribitol | 575497 | 217 | 7362 | C00474 | 827 | 8642 | 8886 | 436 | 198 | 6093 | 10080 |
| raffinose | 1120886 | 361 | 3190 | C00492 | 439242 | 177 | 83 | 230 | 81 | 240 | 38 |
| pyruvic acid | 211668 | 174 | 583 | C00022 | 1060 | 19443 | 2614 | 33161 | 1765 | 33246 | 1121 |
| putrescine | 588872 | 174 | 281 | C00138 | 1049 | 2728 | 1968 | 1854 | 1099 | 2226 | 1573 |
| pseudo uridine | 813899 | 217 | 1688 | C02067 | 15047 | 275 | 146 | 213 | 92 | 257 | 130 |
| proline | 364716 | 142 | 8 | C00148 | 145742 | 397256 | 35967 | 420551 | 36231 | 427464 | 6708 |
| pinitol | 622466 | 260 | 16544 | C03844 | 164619 | 435 | 31 | 328 | 40 | 356 | 39 |
| phosphoethanolamine | 604335 | 299 | 41988 | C00346 | 1015 | 375 | 412 | 131 | 43 | 123 | 80 |
| phenylalanine | 537804 | 218 | 33 | C00079 | 6140 | 67101 | 7435 | 75051 | 5463 | 68071 | 5957 |
| phenylacetic acid | 368081 | 164 | 1733 | C07086 | 999 | 245 | 130 | 214 | 104 | 300 | 121 |
| pentitol | 561721 | 307 | 16624 | D00061 | 827 | 76 | 53 | 63 | 28 | 76 | 40 |
| pelargonic acid | 399229 | 117 | 50 | C01601 | 8158 | 4397 | 663 | 4673 | 1021 | 3664 | 524 |
| p-cresol | 280360 | 165 | 16321 | C01468 | 2879 | 81284 | 6299 | 74724 | 2900 | 75424 | 3303 |
| pantothenic acid | 690887 | 291 | 31356 | C12276 | 6613 | 186 | 37 | 166 | 66 | 150 | 27 |
| palmitic acid | 713809 | 313 | 11 | C00249 | 985 | 8066 | 2421 | 8799 | 1254 | 10042 | 3429 |
| oxoproline | 485935 | 156 | 10 | C01879 | 7405 | 3511425 | 116601 | 3690325 | 225218 | 3663895 | 287988 |
| oxalic acid | 260513 | 190 | 4923 | C00209 | 971 | 1862 | 265 | 1509 | 1256 | 1493 | 163 |
| orotic acid | 586317 | 254 | 18492 | C00295 | 967 | 734 | 781 | 670 | 711 | 650 | 735 |
| ornithine | 527113 | 142 | 1821 | C00077 | 6262 | 299999 | 55591 | 309618 | 41001 | 312902 | 26261 |
| octanol NIST | 247010 | 187 | 49382 |  |  | 325 | 43 | 381 | 53 | 309 | 27 |
| octadecanol | 755409 | 327 | 997 |  | 8221 | 378 | 186 | 378 | 140 | 235 | 77 |
| N-methylalanine | 286855 | 130 | 35 | C02721 | 5288725 | 3833 | 2104 | 1809 | 580 | 1970 | 518 |
| nicotinic acid | 366992 | 180 | 285 | C00253 | 938 | 617 | 66 | 689 | 69 | 569 | 52 |
| n-acetylglutamate | 477776 | 158 | 84199 | C00624 | 70914 | 2354 | 1535 | 3867 | 1327 | 2710 | 1362 |
| n-acetyl-d-hexosamine | 746341 | 319 | 1674 | C03878 | 24139 | 325 | 165 | 254 | 144 | 180 | 68 |
| myristic acid | 634414 | 117 | 127 | C06424 | 11005 | 2805 | 753 | 2620 | 359 | 2921 | 712 |
| myo-inositol | 730022 | 305 | 1741 | C00137 | 892 | 83777 | 1638 | 90683 | 2907 | 90833 | 4012 |
| methionine sulfoxide | 637588 | 128 | 1678 | C02989 | 158980 | 4594 | 2153 | 3511 | 943 | 3689 | 1391 |
| methionine | 483560 | 176 | 45 | C00073 | 6137 | 45918 | 5548 | 44690 | 5796 | 46900 | 3648 |
| maltose | 946601 | 204 | 1979 | C00208 | 439186 | 165 | 59 | 365 | 286 | 260 | 144 |
| malic acid | 463180 | 233 | 1391 | C00711 | 525 | 363 | 146 | 301 | 135 | 283 | 99 |
| maleimide | 245118 | 154 | 1743 | C07272 | 10935 | 437 | 139 | 427 | 40 | 429 | 59 |
| lyxitol | 573587 | 217 | 233 | C00532 | 439255 | 1051 | 359 | 1268 | 507 | 1095 | 438 |
| lysine | 663483 | 156 | 12 | C00047 | 5962 | 346881 | 19332 | 346643 | 30600 | 318030 | 49157 |
| leucine | 346101 | 158 | 9 | C00123 | 6106 | 1346866 | 121155 | 1349014 | 93532 | 1331754 | 88775 |
| lauric acid | 547906 | 117 | 49 | C02679 | 3893 | 3411 | 1406 | 1682 | 915 | 1886 | 973 |
| lactulose | 929908 | 204 | 6432 | C07064 | 11333 | 1005 | 821 | 733 | 549 | 1055 | 941 |
| lactose | 935640 | 191 | 4771 | C00243 | 440995 | 55 | 7 | 49 | 19 | 55 | 15 |
| lactic acid | 217657 | 191 | 80 | C01432 | 612 | 548671 | 197119 | 477829 | 280542 | 529005 | 329663 |
| lactamide | 265698 | 232 | 415 |  | 94220 | 1035 | 409 | 822 | 387 | 836 | 439 |
| itaconic acid | 386511 | 147 | 101725 | C00490 | 811 | 2103 | 744 | 2788 | 423 | 2712 | 1076 |
| isothreonic acid | 489385 | 292 | 1679 | C00639 | 151152 | 1171 | 123 | 1191 | 47 | 1119 | 77 |
| isoleucine | 359251 | 158 | 15 | C00407 | 6306 | 131323 | 4020 | 116854 | 5981 | 112316 | 12475 |
| isocitric acid | 616323 | 245 | 12267 | C00451 | 5318532 | 198 | 28 | 183 | 14 | 195 | 48 |
| inosine | 897434 | 230 | 84524 | C00294 | 6021 | 132 | 99 | 124 | 59 | 139 | 79 |
| hypoxanthine | 619128 | 265 | 1663 | C00262 | 790 | 2688 | 1468 | 1962 | 1032 | 2130 | 1424 |
| hydroxylamine | 255241 | 146 | 139 | C00192 | 787 | 8065 | 1029 | 7443 | 589 | 7447 | 539 |
| homoserine | 443878 | 218 | 2849 | C00263 | 12647 | 637 | 57 | 1152 | 363 | 1107 | 356 |
| histidine | 663790 | 154 | 150 | C00135 | 6274 | 54698 | 31880 | 42023 | 9939 | 57018 | 34127 |
| heptadecanoic acid | 751309 | 117 | 727 |  | 10465 | 799 | 231 | 1090 | 563 | 1505 | 1011 |
| guanine | 744307 | 352 | 2519 | C00242 | 764 | 499 | 292 | 556 | 438 | 559 | 449 |
| gly-pro | 691662 | 373 | 66282 |  | 3013625 | 217 | 120 | 207 | 80 | 189 | 78 |
| glycyl proline | 691357 | 174 | 18496 |  | 3013625 | 1598 | 677 | 1565 | 465 | 1461 | 635 |
| glycolic acid | 227636 | 177 | 1971 | C00160 | 757 | 1939 | 199 | 1563 | 210 | 1534 | 130 |
| glycine | 368707 | 248 | 6 | C00037 | 750 | 37438 | 9175 | 36664 | 4894 | 32700 | 8476 |
| glycerol-alpha-phosphate | 590747 | 357 | 1687 | C03189 | 754 | 72 | 34 | 85 | 37 | 88 | 49 |
| glycerol | 344466 | 205 | 30 | C00116 | 753 | 29938 | 2733 | 53370 | 979 | 41573 | 3377 |
| glyceric acid | 377495 | 189 | 48 | C00258 | 439194 | 3782 | 343 | 3009 | 352 | 3115 | 211 |
| glutamine | 600315 | 156 | 18 | C00064 | 5961 | 576343 | 220181 | 202461 | 163939 | 305799 | 252304 |
| glutamic acid | 529100 | 246 | 28 | C00025 | 33032 | 726532 | 373829 | 1141006 | 102331 | 985858 | 263574 |
| glucose-6-phosphate | 810287 | 387 | 296 | C01172 | 439427 | 113 | 48 | 94 | 25 | 84 | 49 |
| glucose | 659798 | 319 | 22 | C00221 | 64689 | 211763 | 164605 | 222135 | 175358 | 202646 | 168973 |
| gluconic acid | 693148 | 333 | 7501 | C00800 | 6857417 | 36 | 6 | 31 | 11 | 42 | 16 |
| galactinol | 1017580 | 204 | 1975 | C01235 | 11727586 | 651 | 162 | 686 | 69 | 694 | 70 |
| fumaric acid | 390775 | 245 | 1718 | C00122 | 444972 | 408 | 81 | 320 | 70 | 303 | 75 |
| fructose | 639442 | 307 | 21 | C02336 | 439709 | 598 | 198 | 461 | 271 | 344 | 163 |
| erythrose | 443306 | 205 | 102661 | C01796 | 439574 | 167 | 66 | 162 | 43 | 166 | 36 |
| erythritol | 471922 | 217 | 92 | C00503 | 222285 | 1853 | 608 | 1841 | 537 | 1942 | 781 |
| dodecanoic acid, isopropanol ester NIST | 536494 | 200 | 41895 |  |  | 194 | 46 | 169 | 84 | 110 | 27 |
| dehydroabietic acid | 850374 | 239 | 8917 | C12078 | 94391 | 211 | 25 | 232 | 12 | 232 | 52 |
| cystine | 804619 | 218 | 94 | C01420 | 595 | 1794 | 1461 | 1327 | 1034 | 933 | 512 |
| cysteine-glycine | 715335 | 220 | 2787 | C01419 | 439498 | 460 | 281 | 1539 | 683 | 1140 | 611 |
| cysteine | 500158 | 220 | 65 | C00097 | 5862 | 30264 | 5162 | 30022 | 7680 | 32480 | 2614 |
| citrulline | 621404 | 157 | 1712 | C00327 | 9750 | 41656 | 8858 | 34233 | 2519 | 40983 | 3855 |
| citric acid | 617342 | 273 | 288 | C00158 | 311 | 746 | 833 | 92 | 47 | 109 | 69 |
| cholesterol | 1078536 | 129 | 19 | C00187 | 5997 | 261 | 95 | 306 | 122 | 326 | 183 |
| beta-gentiobiose | 973116 | 204 | 100955 | C08240 | 441422 | 1162 | 39 | 1057 | 122 | 1101 | 70 |
| beta-alanine | 435564 | 248 | 148 | C00099 | 239 | 229 | 139 | 189 | 132 | 220 | 98 |
| benzoic acid | 339067 | 179 | 36 | C00180 | 243 | 5417 | 231 | 4162 | 737 | 4107 | 1079 |
| behenic acid | 920648 | 117 | 46315 | C08281 | 8215 | 2054 | 1957 | 708 | 253 | 720 | 352 |
| aspartic acid | 480387 | 232 | 79 | C00049 | 5960 | 9988 | 11750 | 695 | 111 | 746 | 90 |
| asparagine | 553078 | 188 | 146 | C00152 | 6267 | 683 | 118 | 640 | 169 | 622 | 184 |
| aminomalonate | 455754 | 218 | 413 | C00872 | 100714 | 351 | 72 | 307 | 44 | 335 | 119 |
| alpha-ketoglutarate | 507392 | 198 | 294 | C00026 | 51 | 4766 | 2479 | 8112 | 2666 | 6956 | 1455 |
| alanine-alanine | 522546 | 116 | 84119 | C00993 | 5484352 | 2581 | 635 | 4291 | 568 | 3800 | 1491 |
| alanine | 243971 | 116 | 34178 | C00041 | 5950 | 253861 | 58296 | 224549 | 36633 | 246824 | 54491 |
| adenine | 646534 | 264 | 1764 | C00147 | 190 | 88042 | 10519 | 94366 | 3289 | 86626 | 7362 |
| acetophenone NIST | 238615 | 105 | 3524 | C07113 | 7410 | 1966 | 138 | 1584 | 332 | 1556 | 426 |
| 4-aminobutyric acid | 488730 | 304 | 1842 | C00334 | 119 | 264 | 17 | 161 | 35 | 240 | 26 |
| 3-phosphoglycerate | 610734 | 227 | 1667 | C00597 | 724 | 82 | 42 | 139 | 41 | 98 | 8 |
| 2-ketoisocaproic acid | 310761 | 89 | 208 | C00233 | 70 | 7584 | 7427 | 4736 | 4466 | 6423 | 4529 |
| 2-hydroxyglutaric acid | 506306 | 247 | 2000 | C02630 | 43 | 616 | 148 | 560 | 163 | 532 | 49 |
| 2-hydroxybutanoic acid | 258161 | 131 | 40 | C05984 | 440864 | 7228 | 598 | 5554 | 1216 | 5489 | 1109 |
| 2-deoxytetronic acid NIST | 390517 | 117 | 4544 |  | 192742 | 348 | 52 | 117 | 101 | 109 | 72 |
| 2-deoxyerythritol | 354388 | 117 | 34100 |  | 18302 | 334 | 44 | 274 | 19 | 312 | 36 |
| 2,3-dihydroxypyridine | 373895 | 240 | 18043 |  | 28115 | 54 | 16 | 61 | 9 | 54 | 12 |
| 1-monostearin | 959214 | 129 | 648 | D01947 | 24699 | 163 | 126 | 117 | 22 | 162 | 84 |
| 1-monopalmitin | 901749 | 129 | 2070 | C01885 | 14900 | 332 | 284 | 154 | 23 | 184 | 70 |
| 4713 | 939939 | 361 | 4713 |  |  | 47 | 45 | 15 | 10 | 37 | 4 |
| 107960 | 826841 | 158 | 107960 |  |  | 946 | 652 | 872 | 700 | 759 | 702 |
| 107907 | 247953 | 89 | 107907 |  |  | 2556 | 1862 | 1778 | 1053 | 2034 | 1253 |
| 107867 | 219679 | 127 | 107867 |  |  | 4845 | 4425 | 4450 | 3711 | 4757 | 2562 |
| 107143 | 278729 | 174 | 107143 |  |  | 600 | 74 | 567 | 46 | 557 | 10 |
| 107050 | 220203 | 130 | 107050 |  |  | 15445 | 12440 | 7122 | 2171 | 8168 | 2745 |
| 106936 | 238992 | 244 | 106936 |  |  | 515 | 427 | 484 | 170 | 458 | 108 |
| 106935 | 349354 | 140 | 106935 |  |  | 524 | 57 | 545 | 38 | 522 | 76 |
| 106629 | 967520 | 112 | 106629 |  |  | 2646 | 3944 | 400 | 86 | 315 | 320 |
| 106353 | 192179 | 97 | 106353 |  |  | 40424 | 10446 | 30235 | 4480 | 37273 | 5880 |
| 105577 | 273784 | 115 | 105577 |  |  | 7912 | 532 | 6656 | 421 | 6242 | 1032 |
| 105164 | 449834 | 228 | 105164 |  |  | 2061 | 107 | 2109 | 132 | 2140 | 23 |
| 105122 | 370508 | 191 | 105122 |  |  | 23452 | 881 | 22618 | 1800 | 21765 | 1681 |
| 104922 | 729954 | 142 | 104922 |  |  | 1290 | 158 | 1611 | 57 | 1544 | 144 |
| 104901 | 931361 | 204 | 104901 |  |  | 1007 | 820 | 734 | 547 | 1056 | 941 |
| 103886 | 228732 | 165 | 103886 |  |  | 1775 | 1193 | 4534 | 2030 | 2872 | 1261 |
| 103690 | 350735 | 258 | 103690 |  |  | 5372 | 144 | 4914 | 196 | 4664 | 109 |
| 103476 | 475437 | 141 | 103476 |  |  | 77238 | 2029 | 79553 | 1591 | 78078 | 6008 |
| 102978 | 419729 | 95 | 102978 |  |  | 2332 | 74 | 2236 | 32 | 2150 | 127 |
| 102941 | 472321 | 141 | 102941 |  |  | 77265 | 1992 | 79549 | 1586 | 78055 | 6006 |
| 102232 | 551811 | 128 | 102232 |  |  | 2013 | 1676 | 973 | 1016 | 1934 | 1614 |
| 102070 | 456221 | 114 | 102070 |  |  | 2084 | 87 | 1919 | 57 | 1843 | 134 |
| 101742 | 885481 | 117 | 101742 |  |  | 783 | 33 | 524 | 430 | 664 | 138 |
| 101732 | 196225 | 125 | 101732 |  |  | 12111 | 1921 | 11278 | 820 | 11270 | 749 |
| 101035 | 238200 | 119 | 101035 |  |  | 1159 | 179 | 908 | 270 | 959 | 73 |
| 100841 | 837840 | 103 | 100841 |  |  | 7196 | 4788 | 3170 | 2515 | 2667 | 2006 |
| 100768 | 350637 | 188 | 100768 |  |  | 3868 | 168 | 3453 | 117 | 3107 | 567 |
| 100736 | 198538 | 111 | 100736 |  |  | 12100 | 1579 | 11248 | 867 | 11525 | 613 |
| 100730 | 280637 | 171 | 100730 |  |  | 23840 | 1483 | 23203 | 1723 | 18747 | 5561 |
| 100584 | 502056 | 128 | 100584 |  |  | 13045 | 336 | 12543 | 618 | 12062 | 737 |
| 100326 | 710069 | 258 | 100326 |  |  | 367 | 257 | 408 | 123 | 263 | 77 |
| 100017 | 844931 | 217 | 100017 |  |  | 219 | 150 | 193 | 139 | 130 | 39 |
| 98027 | 794158 | 217 | 98027 |  |  | 265 | 85 | 219 | 101 | 268 | 121 |
| 97891 | 932190 | 156 | 97891 |  |  | 360 | 230 | 84 | 24 | 149 | 102 |
| 96539 | 721995 | 156 | 96539 |  |  | 689 | 882 | 734 | 1071 | 753 | 1039 |
| 95422 | 572506 | 100 | 95422 |  |  | 7407 | 916 | 8161 | 2884 | 10335 | 3701 |
| 91421 | 280725 | 86 | 91421 |  |  | 237414 | 41269 | 232443 | 58790 | 199279 | 16315 |
| 87877 | 330723 | 110 | 87877 |  |  | 1629 | 157 | 1425 | 62 | 1268 | 39 |
| 87282 | 444113 | 103 | 87282 |  |  | 886 | 238 | 1353 | 209 | 1345 | 83 |
| 85170 | 938000 | 202 | 85170 |  |  | 65 | 32 | 44 | 11 | 51 | 28 |
| 84583 | 631741 | 156 | 84583 |  |  | 2319 | 1410 | 3857 | 1389 | 3165 | 2259 |
| 64546 | 699999 | 204 | 64546 |  |  | 22912 | 17779 | 24456 | 18871 | 21906 | 19678 |
| 62409 | 303304 | 170 | 62409 |  |  | 475 | 50 | 430 | 32 | 457 | 25 |
| 54643 | 591742 | 197 | 54643 |  |  | 1029 | 121 | 928 | 155 | 919 | 562 |
| 53724 | 594337 | 197 | 53724 |  |  | 961 | 184 | 908 | 132 | 1100 | 531 |
| 47286 | 511792 | 185 | 47286 |  |  | 929 | 298 | 479 | 114 | 558 | 227 |
| 46404 | 915863 | 117 | 46404 |  |  | 589 | 228 | 713 | 244 | 643 | 398 |
| 43580 | 798275 | 117 | 43580 |  |  | 2473 | 748 | 1792 | 742 | 2153 | 1150 |
| 43100 | 414838 | 88 | 43100 |  |  | 1454 | 414 | 1760 | 482 | 1511 | 256 |
| 42205 | 590106 | 197 | 42205 |  |  | 461 | 80 | 417 | 61 | 512 | 123 |
| 41938 | 272954 | 157 | 41938 |  |  | 3907 | 313 | 3579 | 112 | 3250 | 167 |
| 41821 | 445721 | 243 | 41821 |  |  | 244 | 11 | 273 | 203 | 570 | 412 |
| 41811 | 568579 | 197 | 41811 |  |  | 362 | 28 | 308 | 85 | 460 | 127 |
| 41808 | 572340 | 197 | 41808 |  |  | 430 | 185 | 453 | 80 | 585 | 193 |
| 33415 | 319800 | 156 | 33415 |  |  | 942 | 124 | 803 | 52 | 771 | 76 |
| 33386 | 496938 | 219 | 33386 |  |  | 1485 | 330 | 2101 | 113 | 1898 | 42 |
| 26924 | 1029169 | 295 | 26924 |  |  | 6325 | 10730 | 345 | 431 | 229 | 278 |
| 26746 | 882083 | 105 | 26746 |  |  | 983 | 672 | 589 | 75 | 607 | 38 |
| 23635 | 308124 | 85 | 23635 |  |  | 986 | 269 | 1019 | 181 | 914 | 235 |
| 22423 | 230574 | 170 | 22423 |  |  | 1079 | 289 | 833 | 163 | 880 | 272 |
| 21704 | 983235 | 204 | 21704 |  |  | 388 | 114 | 407 | 175 | 414 | 122 |
| 18488 | 456907 | 232 | 18488 |  |  | 369 | 70 | 309 | 22 | 343 | 10 |
| 18225 | 476014 | 179 | 18225 |  |  | 784 | 31 | 818 | 61 | 783 | 64 |
| 18173 | 415283 | 131 | 18173 |  |  | 311 | 55 | 308 | 79 | 274 | 110 |
| 17664 | 227179 | 173 | 17664 |  |  | 819 | 106 | 672 | 103 | 604 | 291 |
| 17651 | 535097 | 275 | 17651 |  |  | 276 | 32 | 196 | 94 | 270 | 3 |
| 17267 | 562200 | 243 | 17267 |  |  | 604 | 234 | 349 | 147 | 568 | 403 |
| 17253 | 557417 | 252 | 17253 |  |  | 271 | 90 | 332 | 82 | 276 | 121 |
| 17252 | 450982 | 128 | 17252 |  |  | 12774 | 5742 | 7217 | 2188 | 12228 | 2246 |
| 17245 | 280956 | 174 | 17245 |  |  | 600 | 74 | 556 | 27 | 557 | 10 |
| 17068 | 265535 | 267 | 17068 |  |  | 882 | 138 | 756 | 199 | 807 | 86 |
| 17011 | 883952 | 204 | 17011 |  |  | 107 | 58 | 87 | 53 | 81 | 34 |
| 16817 | 758926 | 174 | 16817 |  |  | 50 | 23 | 146 | 26 | 104 | 36 |
| 16788 | 865160 | 446 | 16788 |  |  | 2291 | 1990 | 2354 | 2100 | 2326 | 2428 |
| 16747 | 742150 | 319 | 16747 |  |  | 412 | 116 | 421 | 112 | 412 | 200 |
| 16567 | 967921 | 204 | 16567 |  |  | 648 | 27 | 440 | 341 | 234 | 343 |
| 16561 | 245883 | 155 | 16561 |  |  | 1907 | 58 | 1703 | 41 | 1698 | 76 |
| 13107 | 545737 | 243 | 13107 |  |  | 737 | 399 | 324 | 224 | 464 | 429 |
| 10962 | 336339 | 249 | 10962 |  |  | 513 | 118 | 491 | 87 | 356 | 68 |
| 9489 | 688397 | 156 | 9489 |  |  | 518 | 57 | 536 | 22 | 448 | 96 |
| 9320 | 705438 | 91 | 9320 |  |  | 3846 | 699 | 1164 | 733 | 1438 | 1448 |
| 7490 | 466348 | 160 | 7490 |  |  | 1970 | 321 | 1077 | 595 | 1387 | 411 |
| 6104 | 241903 | 244 | 6104 |  |  | 699 | 110 | 659 | 121 | 705 | 117 |
| 5990 | 301233 | 188 | 5990 |  |  | 287 | 30 | 327 | 97 | 274 | 101 |
| 5576 | 300553 | 197 | 5576 |  |  | 372 | 26 | 402 | 160 | 321 | 126 |
| 5346 | 494094 | 85 | 5346 |  |  | 2251 | 222 | 2054 | 272 | 2288 | 73 |
| 5290 | 928811 | 132 | 5290 |  |  | 2692 | 1820 | 2636 | 2070 | 2419 | 2111 |
| 5085 | 799884 | 98 | 5085 |  |  | 9155 | 5953 | 4319 | 3568 | 2978 | 2413 |
| 4937 | 1200184 | 316 | 4937 |  |  | 328 | 42 | 274 | 139 | 351 | 6 |
| 4746 | 548258 | 275 | 4746 |  |  | 5577 | 2067 | 2724 | 773 | 3221 | 1738 |
| 4546 | 782748 | 217 | 4546 |  |  | 1099 | 415 | 925 | 612 | 887 | 721 |
| 4531 | 271256 | 262 | 4531 |  |  | 597 | 28 | 703 | 94 | 462 | 139 |
| 4086 | 194874 | 147 | 4086 |  |  | 14372 | 6639 | 7564 | 1544 | 7806 | 3126 |
| 3465 | 564741 | 129 | 3465 |  |  | 904 | 152 | 576 | 193 | 524 | 256 |
| 3442 | 474455 | 141 | 3442 |  |  | 77256 | 1991 | 79553 | 1591 | 78079 | 6008 |
| 3328 | 802998 | 85 | 3328 |  |  | 2592 | 235 | 521 | 239 | 640 | 621 |
| 3258 | 265343 | 151 | 3258 |  |  | 1148 | 224 | 1389 | 148 | 1448 | 207 |
| 3232 | 952057 | 204 | 3232 |  |  | 1742 | 105 | 1613 | 173 | 1627 | 126 |
| 3143 | 459926 | 155 | 3143 |  |  | 7351 | 7759 | 585 | 506 | 8177 | 10013 |
| 3086 | 202796 | 111 | 3086 |  |  | 3275 | 500 | 3128 | 168 | 2905 | 627 |
| 3085 | 202456 | 97 | 3085 |  |  | 4007 | 816 | 3286 | 662 | 3602 | 386 |
| 3082 | 203562 | 85 | 3082 |  |  | 7160 | 1266 | 6003 | 1237 | 6798 | 455 |
| 2944 | 214208 | 97 | 2944 |  |  | 12738 | 1851 | 11741 | 813 | 11898 | 1014 |
| 2900 | 569952 | 156 | 2900 |  |  | 3117 | 940 | 3240 | 544 | 2664 | 1090 |
| 2510 | 762401 | 98 | 2510 |  |  | 196 | 61 | 50 | 16 | 103 | 96 |
| 2439 | 542827 | 102 | 2439 |  |  | 547 | 68 | 569 | 145 | 561 | 125 |
| 2189 | 475636 | 230 | 2189 |  |  | 757 | 154 | 758 | 182 | 643 | 189 |
| 2097 | 254762 | 85 | 2097 |  |  | 1440 | 460 | 1495 | 66 | 1509 | 143 |
| 2095 | 230280 | 123 | 2095 |  |  | 230 | 58 | 225 | 18 | 203 | 99 |
| 2081 | 242781 | 97 | 2081 |  |  | 1098 | 283 | 923 | 155 | 933 | 58 |
| 2061 | 250126 | 85 | 2061 |  |  | 2148 | 274 | 1891 | 8 | 1794 | 197 |
| 2037 | 756085 | 290 | 2037 |  |  | 790 | 608 | 837 | 385 | 751 | 575 |
| 2031 | 632838 | 245 | 2031 |  |  | 2083 | 185 | 2115 | 6 | 2001 | 79 |
| 1996 | 320124 | 156 | 1996 |  |  | 942 | 121 | 800 | 47 | 770 | 80 |
| 1981 | 422675 | 103 | 1981 |  |  | 3146 | 87 | 2904 | 29 | 2906 | 159 |
| 1969 | 669949 | 336 | 1969 |  |  | 982 | 159 | 1062 | 286 | 865 | 251 |
| 1913 | 307995 | 130 | 1913 |  |  | 756 | 419 | 355 | 83 | 473 | 114 |
| 1912 | 855692 | 187 | 1912 |  |  | 574 | 49 | 544 | 13 | 586 | 30 |
| 1875 | 592702 | 85 | 1875 |  |  | 1325 | 172 | 1225 | 107 | 1475 | 239 |
| 1831 | 718162 | 257 | 1831 |  |  | 187 | 34 | 194 | 14 | 166 | 34 |
| 1826 | 872787 | 156 | 1826 |  |  | 11256 | 8691 | 11937 | 10166 | 11214 | 11828 |
| 1771 | 527322 | 166 | 1771 |  |  | 1097 | 377 | 1515 | 445 | 1083 | 145 |
| 1753 | 795138 | 290 | 1753 |  |  | 380 | 289 | 369 | 215 | 328 | 279 |
| 1746 | 766150 | 156 | 1746 |  |  | 929 | 81 | 935 | 81 | 845 | 18 |
| 1742 | 720191 | 116 | 1742 |  |  | 949 | 180 | 845 | 276 | 696 | 390 |
| 1732 | 686299 | 142 | 1732 |  |  | 440 | 154 | 326 | 49 | 323 | 51 |
| 1725 | 627764 | 120 | 1725 |  |  | 2645 | 341 | 1332 | 542 | 1479 | 673 |
| 1721 | 844722 | 156 | 1721 |  |  | 826 | 332 | 904 | 217 | 699 | 177 |
| 1713 | 717217 | 156 | 1713 |  |  | 2155 | 142 | 1884 | 207 | 1768 | 177 |
| 1711 | 555956 | 138 | 1711 |  |  | 463 | 70 | 454 | 179 | 454 | 55 |
| 1709 | 315703 | 212 | 1709 |  |  | 11835 | 427 | 10463 | 563 | 9811 | 342 |
| 1708 | 759197 | 156 | 1708 |  |  | 2661 | 399 | 2617 | 174 | 2254 | 257 |
| 1704 | 505341 | 223 | 1704 |  |  | 667 | 158 | 830 | 219 | 584 | 174 |
| 1702 | 677891 | 156 | 1702 |  |  | 1945 | 326 | 1811 | 161 | 1602 | 148 |
| 1684 | 743774 | 262 | 1684 |  |  | 67 | 56 | 47 | 29 | 84 | 48 |
| 1665 | 192579 | 168 | 1665 |  |  | 2871 | 575 | 2033 | 115 | 1394 | 437 |
| 1380 | 294677 | 103 | 1380 |  |  | 7645 | 2728 | 6695 | 2473 | 4757 | 994 |
| 1173 | 239709 | 151 | 1173 |  |  | 984 | 405 | 1235 | 102 | 1248 | 110 |
| 1148 | 189922 | 147 | 1148 |  |  | 12522 | 9023 | 6182 | 2463 | 7982 | 3426 |
| 1064 | 594945 | 117 | 1064 |  |  | 7556 | 947 | 4275 | 1619 | 5104 | 1281 |
| 892 | 478262 | 155 | 892 |  |  | 1326 | 1301 | 4768 | 2548 | 2462 | 436 |
| 782 | 571259 | 304 | 782 |  |  | 496 | 150 | 2687 | 638 | 2116 | 191 |
| 657 | 227730 | 173 | 657 |  |  | 800 | 137 | 678 | 109 | 579 | 256 |
| 592 | 514932 | 142 | 592 |  |  | 2156 | 513 | 2598 | 546 | 2273 | 920 |
| 490 | 336870 | 157 | 490 |  |  | 7877 | 1143 | 5526 | 984 | 6074 | 810 |
| 479 | 472741 | 98 | 479 |  |  | 7091 | 1868 | 10871 | 523 | 8402 | 3424 |
| 473 | 589773 | 193 | 473 |  |  | 1163 | 128 | 1346 | 117 | 1144 | 155 |
| 462 | 341008 | 259 | 462 |  |  | 428 | 41 | 314 | 19 | 319 | 29 |
| 453 | 280176 | 86 | 453 |  |  | 237414 | 41128 | 232296 | 58476 | 199066 | 16224 |
| 307 | 637461 | 174 | 307 |  |  | 9472 | 1999 | 15991 | 7457 | 17314 | 11107 |
| 299 | 430778 | 104 | 299 |  |  | 9999 | 4486 | 11716 | 2486 | 9027 | 1102 |
| 257 | 1134576 | 441 | 257 |  |  | 157 | 56 | 163 | 35 | 179 | 16 |
| 228 | 413558 | 191 | 228 |  |  | 1104 | 397 | 668 | 140 | 593 | 58 |
| 168 | 878989 | 122 | 168 |  |  | 635 | 694 | 165 | 26 | 124 | 149 |
| 137 | 246162 | 204 | 137 |  |  | 1184 | 277 | 1062 | 233 | 900 | 215 |
| 136 | 579727 | 227 | 136 |  |  | 219916 | 173984 | 65592 | 87423 | 169749 | 212590 |
| 134 | 974612 | 144 | 134 |  |  | 3854 | 5229 | 1492 | 401 | 1328 | 1652 |
| 110 | 298570 | 132 | 110 |  |  | 496 | 132 | 400 | 59 | 391 | 41 |
| 91 | 221732 | 100 | 91 |  |  | 3277 | 587 | 3078 | 207 | 2945 | 221 |
| 68 | 195282 | 89 | 68 |  |  | 473 | 257 | 222 | 115 | 190 | 107 |
| 62 | 247887 | 85 | 62 |  |  | 4392 | 197 | 2122 | 1337 | 3745 | 383 |
| 61 | 511029 | 185 | 61 |  |  | 931 | 298 | 479 | 114 | 557 | 225 |
| 47 | 354110 | 85 | 47 |  |  | 4577 | 155 | 3952 | 45 | 4160 | 141 |

Supplementary Table 2. The primers used in Quantitative Real-Time PCR analysis

| Species | Gene | Primer |
| --- | --- | --- |
| Human | interleukin 1 alpha (IL-1α) | Hs_IL1A_1_SG QuantiTect Primer Assay |
|  | interleukin 6 (IL-6) | Hs_IL6_1_SG QuantiTect Primer Assay |
|  | interleukin 18 (IL-18) | Hs_IL18_1_SG QuantiTect Primer Assay |
|  | caspase 3 (CASP3) | Hs_CASP3_1_SG QuantiTect Primer Assay |
|  | matrix metallopeptidase 1 (MMP-1) | Hs_MMP1_1_SG QuantiTect Primer Assay |
|  | tumor necrosis factor (TNF) | Hs_TNF_3_SG QuantiTect Primer Assay |
